# Informed circular fields: a global reactive obstacle avoidance framework for robotic manipulators

**DOI:** 10.3389/frobt.2024.1447351

**Published:** 2025-01-03

**Authors:** Marvin Becker, Philipp Caspers, Torsten Lilge, Sami Haddadin, Matthias A. Müller

**Affiliations:** ^1^ Institute of Automatic Control, Leibniz University Hannover, Hannover, Germany; ^2^ Munich Institute of Robotics and Machine Intelligence, Technische Universität München (TUM), Munich, Germany

**Keywords:** autonomous robotic systems, guidance navigation and control, real-time collision avoidance, robotic manipulation arm, motion planning

## Abstract

In this paper, we present a global reactive motion planning framework designed for robotic manipulators navigating in complex dynamic environments. Utilizing local minima-free circular fields, our methodology generates reactive control commands while also leveraging global environmental information from arbitrary configuration space motion planners to identify promising trajectories around obstacles. Furthermore, we extend the virtual agents framework introduced in Becker et al. (2021) to incorporate this global information, simulating multiple robot trajectories with varying parameter sets to enhance avoidance strategies. Consequently, the proposed unified robotic motion planning framework seamlessly combines global trajectory planning with local reactive control and ensures comprehensive obstacle avoidance for the entire body of a robotic manipulator. The efficacy of the proposed approach is demonstrated through rigorous testing in over 4,000 simulation scenarios, where it consistently outperforms existing motion planners. Additionally, we validate our framework’s performance in real-world experiments using a collaborative Franka Emika robot with vision feedback. Our experiments illustrate the robot’s ability to promptly adapt its motion plan and effectively avoid unpredictable movements by humans within its workspace. Overall, our contributions offer a robust and versatile solution for global reactive motion planning in dynamic environments.

## 1 Introduction

### 1.1 Motivation

In recent years, the prerequisites of industrial production and assembly have changed significantly and the ensuing challenges are constantly evolving. Continuously decreasing product life cycles, uncertain product volumes and rapidly growing product variants due to the growing trend towards mass customization have led to an increasing demand for flexible, adaptive multi-purpose manufacturing and assembly systems (El Zaatari et al., 2019; Krüger et al., 2009; Reinhart and Loy, 2010; Fechter et al., 2016).

Although classical industrial robots have proven to be efficient tools for repeatable tasks in traditional mass production, they need to be operated behind safety fences in dedicated areas to ensure the safety of human coworkers, which limits their flexibility and reusability while increasing their changeover times, costs and space requirements (Matheson et al., 2019; Barbazza et al., 2017).

Human robot interaction (HRI) is generally seen as a promising solution to increase flexibility while reducing production and assembly costs in continuously changing and uncertain market environments (Matheson et al., 2019; Kragic et al., 2018; Fechter et al., 2016; Krüger et al., 2009; El Zaatari et al., 2019; Faccio et al., 2019; Meziane et al., 2017). However, despite the widely recognized potential and the long-standing availability of the technology, the use of collaborative systems is still almost exclusively limited to areas where no direct contact with humans is necessary and the workspaces are structurally separated from each other. Moreover, robots employed in industry typically adhere to rigidly pre-programmed trajectories and routines and possess only limited capabilities to interact or react to changing environmental conditions (Kragic et al., 2018; El Zaatari et al., 2019).

To cope with the challenges in the dynamic and unpredictable environments around humans, robots must be able to adapt quickly to changing conditions and generate new trajectories in real time (Kappler et al., 2018; Liu et al., 2022).

Collision avoidance is a key component for solving such motion planning problems and while a lot of research has been conducted in this field, challenges persist, in particular in dynamic environments Huang et al. (2019); Niloy et al. (2021).

### 1.2 Related work

The subsequent overview of the related work is organized by following the allocation from (Kappler et al., 2018), which classifies motion planning approaches into the categories *sense-plan-act*, *locally reactive control* and *reactive planning*.

System architectures that follow the *sense-plan-act* paradigm are characterized by a rather strict separation of perception, motion planning and control. Typically, sensor feedback is considered at the initial stage to update an environment model. The motion planner then uses this model to identify a preliminary, coarse path towards the goal. Subsequently, a separate controller is employed to track this collision-free path (Kappler et al., 2018). Due to the vast amount of research in this field, we focus our review on sampling-based planning approaches in this category.

Sampling-based planners typically establish a connectivity graph between the initial and goal configurations of the robot by connecting random samples in the search space. The concept received significant attention in the field of motion planning over the last decades due to its ability to handle high degrees of freedom, and its straightforward implementation (Connell and La, 2018).

The rapidly-exploring random trees (RRT) (LaValle, 1998) and the probabilistic roadmap method (PRM) (Kavraki et al., 1996) are two of the most commonly used sampling-based approaches in robotics (Short et al., 2016).

The RRT algorithm incrementally constructs a search tree in the robot’s configuration space to find a feasible path to the goal pose starting from the initial robot configuration. A disadvantage of the RRT planner and its variants is the lack of quality in terms of path length, which led to the development of an asymptotically optimal variant, the RRT* planner (Karaman and Frazzoli, 2011). Given enough run-time, the RRT* was shown to converge to an optimal solution by rewiring the search tree and continuously adding new nodes even after an initial solution was found.

In contrast to RRT approaches, the PRM algorithm consists of two phases, a learning phase and a query phase. During the learning phase, the configuration space of the robot is randomly sampled and robot configurations that collide with known obstacles in the task space are rejected. Afterwards, the resulting roadmap is used as a basis for the query phase, where the shortest path from a start to a goal pose is calculated (Kavraki et al., 1996). Similar to RRT, an asymptotically optimal variant, the PRM* was developed, which led to significantly improved path quality (Karaman and Frazzoli, 2011).

Despite recent advances and efficient implementations with replanning capabilities, such as the RRT*FN-Dynamic (RRT*FND) planner (Adiyatov and Varol, 2017), Batch Informed Trees (BIT*) (Gammell et al., 2020), the Bidirectional Informed RRT* (BI^2^RRT*) (Burget et al., 2016) and a dynamically replanned RRT* (Connell and La, 2017), sampling-based approaches still face significant challenges. They tend to have high computational costs (Grothe et al., 2022) and often require post-processing steps to smooth and shorten the generated trajectories (Schulman et al., 2014). Additionally, their performance can degrade notably when navigating through narrow passages (Li and Dantam, 2023).

A seminal work in the area of *locally reactive control* is the artificial potential field (APF) approach, where the robot is controlled by artificial repulsive and attractive forces for a collision-free motion to the goal pose (Khatib, 1986). While the algorithm requires low computational resources, it suffers from local minima. This can cause the robot to converge towards them instead of reaching the goal pose, depending on the environment. Many variants of APFs or related approaches were proposed to overcome this limitation and to enable goal convergence in a wider range of applications, notably the harmonic potential functions (Connolly et al., 1990), which was further extended by Khansari-Zadeh and Billard (2012), and successfully used to avoid static and dynamic obstacles with the endeffector (EE) of a 7-degree of freedom (DoF) manipulator. Related methods use repulsive forces or velocities for collision avoidance of the whole structure of the robot, which were either applied on predefined control points along the robot structure (Chen and Song, 2018; Li et al., 2019) or use the closest distance between robot and obstacle (Wang et al., 2018). Further extensions exploit the robot’s nullspace in order to achieve collision avoidance and simultaneously maintain a predefined EE trajectory, e.g., the approach from Cefalo et al. (2017), where a focus is placed on parallel computation and collision checks on a GPU. Similarly, the approach by (Flacco et al., 2012) developed a fast method for the minimal distance calculation, which was employed to determine repulsive forces for collision avoidance and successfully implemented on a 7-DoF robot.

Inspired by the behavior of charged particles in electromagnetic fields, the authors in (Singh et al., 1996; Singh et al., 1997) developed the circular field (CF) approach. CFs apply a virtual force similar to the Lorentz force on the robot, which results in smooth trajectories around obstacles. The virtual force does not induce any additional energy into the system as it always acts perpendicular to the robot’s velocity and thus does not suffer from local minima. The original algorithm was extended in Haddadin et al. (2011) as it suffered from oscillations due to inconsistently defined artificial currents. Therefore, a rotation vector is introduced for each obstacle in order to define a consistent artificial current flow for each obstacle. An alternative approach was presented in a series of works (Ataka et al., 2018a; Ataka et al., 2018b; Ataka et al., 2018c; Ataka et al., 2022), enabling the algorithm to be used in unknown environments. This is achieved by projecting the robot’s velocity vector onto the obstacle to define a continuous artificial current, thus avoiding the calculation of the rotation vector.

Nevertheless, current CF and APF approaches can only serve as local planners and perform poorly for finding global optimal or even suboptimal solutions because of their limited exploration possibilities.

*Reactive planners* are hybrid motion planners designed to respond quickly to local changes while improving and correcting the global path in response to larger environmental changes (Kappler et al., 2018). Such hybrid approaches have been extensively studied in the literature of mobile robotics and unmanned aerial vehicles (UAV). Implementations often combine PRM and APF (Ravankar et al., 2020), RRT and APF (Yingqi et al., 2021) or variations of existing sampling-based strategies with custom reactive controllers, e.g., lazy PRM with a reactive controller for dynamic obstacles (Sánchez et al., 2006) or RRT-connect with a reactive control law employing a sliding mode control scheme (Elmokadem and Savkin, 2021). 

 While these hybrid planners work effectively in lower DoF mobile robotic systems, their applications to systems with higher DoF, such as robotic manipulators, are relatively sparse (Kappler et al., 2018). For example, (Liu and Jiang, 2018) proposes an approach that alternates between RRT for static obstacles and an APF variant for dynamic obstacles, while (Li et al., 2021) deform the trajectory of an RRT planner using locally reactive control for a 7-DoF manipulator. Similarly, the elastic strip framework (Brock and Khatib, 2002) modifies global candidate paths using APF for real-time reactions to environmental changes.

The authors in Kappler et al. (2018) use global Riemannian motion optimization and locally reactive control for several experiments with a 7-DoF robot arm. In their work, they conduct a comprehensive comparison of *sense-plan-act* methods, *locally reactive control* and *reactive planning*. Their findings suggest that reactive methods have distinct advantages over traditional approaches, particularly in dynamic and uncertain environments. Even in static environments, *reactive planners* showed competitive, and sometimes superior, performance over traditional planning methods, due to their ability to initiate movement without excessive pre-planning. *Reactive planners*, by integrating global information, provide superior adaptability in complex environments compared to purely local methods.

However, a significant limitation of these existing hybrid approaches is the strict separation between global and local planning. In such methods, the global planner and local planner are typically treated as distinct modules, with the local planner often following the global planner’s path without fully leveraging the global insights. This can lead to inefficient planning, where local reactivity may disrupt global goals, or the global planner’s computational cost is prohibitive in dynamic environments.

In our previous work Becker et al. (2021), we introduced the circular field predictions (CFP) method, which enhanced the original CF approach by incorporating a predictive virtual agent framework for global path exploration. The CFP planner demonstrated computational efficiency and high-quality path generation on a 7-DoF manipulator. In subsequent work Becker et al. (2023b), we provided rigorous proofs of collision avoidance and goal convergence, validating the theoretical soundness of the approach. However, these efforts were primarily focused on end-effector obstacle avoidance and did not fully explore full-body obstacle avoidance for manipulators.

This paper builds on these previous efforts by extending the CFP planner to handle full-body obstacle avoidance. Unlike traditional methods, which often focus only on the end-effector or parts of the robot, our approach ensures that the entire manipulator avoids obstacles while maintaining smooth, collision-free trajectories. This full-body approach is particularly useful in environments where dynamic and cluttered obstacles pose significant challenges to the whole body of the robot.

One significant advantage of our approach over existing hybrid planners is the efficient use of global planning insights within the local planning strategy. By avoiding the strict separation between global and local planning found in other methods, our approach ensures that global information is dynamically leveraged in real-time, leading to more efficient and smooth obstacle avoidance. This allows the local reactive controller to not just follow the global path but to dynamically adjust the trajectory based on the global strategy. Moreover, our motion planning strategy is flexible enough to integrate arbitrary global planners, allowing the framework to adapt to different problem settings and robot configurations.

In summary, traditional motion planning methods, while effective in handling high DoF systems, often face significant computational challenges, especially in dynamic environments. Reactive planners provide advantages in real-time reactivity but struggle with global optimization and goal convergence. The proposed approach presents a hybrid planning framework that balances global trajectory planning with local reactivity, ensuring full-body obstacle avoidance in highly dynamic and uncertain environments. This novel approach extends the state of the art in motion planning for robotic manipulators, offering a scalable, computationally efficient solution for future robotic applications in complex environments.

### 1.3 Contribution

In this paper, we extend the CFP algorithm to encompass full-body avoidance for the entire structure of a robotic manipulator, thereby developing a comprehensive robotic motion planning framework that bridges the gap between global trajectory planning and reactive control. The main contributions of this paper are.

• Development of a motion planning framework for full-body obstacle avoidance of robotic manipulators by introducing additional control points along the robot structure and defining suitable control forces.

• Integration of global environment information from arbitrary global planners in the virtual agent framework from (Becker et al., 2021) resulting in the informed circular field (ICF) planner.

• Two algorithms for leveraging global information about promising avoidance directions with the reactive ICF planner.

• The performance of 20 different global planners within the framework is compared in 10 different environments with a total of 4,000 simulations.

• Extensive comparison of the ICF planner against widely used global and local motion planning approaches in a total of more than 200 simulations.

• Verification of the proposed algorithm in real-world experiments, where a 7-DoF Franka Emika Research 3 robot avoids dynamic motions of a human in its workspace.

• Making the planning framework available for the community by providing the source code^1^.

#### 1.3.1 Relationship to previous publications

Note that a preliminary version of parts of this paper has appeared in the conference paper (Becker et al., 2023a). In contrast to our prior work, this article provides a more thorough description of the planner details. Specifically, we expand on the motivation and related work section, provide a detailed description of the calculations for all forces and methods involved in generating robot control commands, and explain the extension of the virtual agent framework, including the new reward function and its structure in detail–elements that had to be omitted in Becker et al. (2023a) due to space limitations. Furthermore, we extend the methodology for the robot control signal calculation, provide an open-source implementation of our code base, and conduct additional extensive simulations to determine appropriate global pre-planners. Lastly, we implement the planning framework on a 7-DoF Franka Emika robot, integrating a vision system for the detection and tracking of humans within the robot workspace for performing real-world experiments to demonstrate the efficacy of the motion planning framework.

## 2 Motion planning framework

In this section, we introduce our unified motion planning framework that consists of a reactive CF obstacle avoidance algorithm inspired by electromagnetic fields combined with a global motion planning approach. The global planning component leverages the results from existing joint space motion planners by extracting information about potential avoidance directions around obstacles, which are subsequently exploited by a virtual agent framework for efficient global exploration.

### 2.1 Reactive motion planning

We start the description of the reactive planning component with an introduction to the steering forces acting on the EE of the robot. Then, we continue with a presentation of the forces for full-body obstacle avoidance and additional forces and supplementary methods for enhancing the overall motion planning strategy of robotic manipulators. The combination of all control commands for generating a total reference signal for joint space control is described in Section 2.1.9.

Our reactive vector-field approach is based on the definition of several virtual forces to generate efficient, collision-free trajectories, which are superimposed to form the resulting steering force $f_{s}$ for controlling the EE

$$f_{s}=f_{vlc}+f_{cf}+f_{cf,rep}.$$ (1)

It consists of an attractive potential field force $f_{vlc}$ for goal convergence and CF-based obstacle avoidance forces $f_{cf}$ and $f_{cf,rep}$ , which are explained in detail in the following sections.

#### 2.1.1 Attractive goal force

In order to guide the robot EE to its goal pose, we extend the definition of potential field attractor dynamics with the proposed velocity limiting controller (VLC) from Khatib (1986). For this purpose, we define an artificial desired velocity in the form

$$v_{\text{d}}=\frac{k_{p}}{k_{v}}\left(x_{g}-x\right),$$

where $x\in R^{3}$ is the current robot position, $x_{g}\in R^{3}$ is the translational part of the goal pose, $k_{p}>0$ is the position gain and $k_{v}>0$ the velocity gain. Note that the orientation is considered separately below. The virtual attractive force $f_{vlc,t}$ is then calculated from the difference of the current robot velocity $\dot{x}$ and the artificial desired velocity $v_{\text{d}}$

$$f_{vlc,t}=k_{v}\left(\nu v_{\text{d}}-\dot{x}\right).$$

The factor ν is used to limit the force when the robot velocity reaches a defined maximum magnitude ${\dot{x}}_{max}$ in the direction of the goal

$$\nu =min\left(1,\frac{{\dot{x}}_{max}}{\left|\;|v_{\text{d}}|\;\right|}\right).$$

Compared to a classical potential field, the resulting control law is more appropriate for longer distances between the robot and the goal pose. In fact, the generated virtual force is equal to zero when the robot moves towards the goal pose at maximum velocity [as shown in (Becker et al., 2023b)]. This leads to a constant velocity magnitude except in the vicinity of the start, goal and obstacles when the robot is subject to further virtual forces. Note that while the VLC controller explicitly enforces a velocity limit, the acceleration limit is only implicitly considered in the current formulation, and it is not directly constrained. Furthermore, even though the control force is restricted by the velocity limit, the gains $k_{p}$ and $k_{v}$ should still be selected with caution to avoid undesirable dynamic behaviors such as oscillations or overshooting. The VLC controller functions similarly to a proportional-derivative (PD) controller, where the balance between these gains is critical. In particular, to achieve stable and smooth control, the gains should be chosen to approximate critical damping in a PD-control scheme, which typically requires setting $k_{v}\approx 2\sqrt{k_{p}}$ . By choosing $k_{p}$ and $k_{v}$ carefully, the robot can maintain smooth and stable motion even in challenging scenarios, while adhering to the velocity limits imposed by the controller.

We extend the approach to include an additional rotation for the robot to achieve the desired orientation $x_{g,r}$ represented in Euler angles. To do this, we define an artificial desired angular velocity $\omega_{\text{d}}$ based on the orientation error $x_{g,r}-x_{r}$ . This error is calculated using the difference between the quaternion representing the robot’s current orientation, $p={\left(\begin{array}{cc} p_{0} & p_{\text{im}}^{⊤} \end{array}\right)}^{⊤}={\left(\begin{array}{cccc} p_{0} & p_{1} & p_{2} & p_{3} \end{array}\right)}^{⊤}$ , and the quaternion for the desired goal orientation, $p_{g}={\left(\begin{array}{cc} p_{g,0} & p_{\text{g,im}}^{⊤} \end{array}\right)}^{⊤}={\left(\begin{array}{cccc} p_{g,0} & p_{g,1} & p_{g,2} & p_{g,3} \end{array}\right)}^{⊤}$ , as described by Yuan, (1988)

$$x_{g,r}-x_{r}=p_{0}p_{\text{g,im}}-p_{g,0}p_{\text{im}}-p_{\text{im}}\times p_{\text{g,im}}.$$

In this formulation, $p_{0}$ and $p_{g,0}$ are the scalar components, while $p_{\text{im}}$ and $p_{\text{g,im}}$ represent the vector (imaginary) components of the quaternions describing the robot’s current and desired orientations, respectively.

The resulting rotational component is then calculated with.

$$\omega_{\text{d}}=\frac{k_{p}}{k_{v}}\left(x_{g,r}-x_{r}\right),$$

$$\nu_{\text{r}}=min\left(1,\frac{\omega_{max}}{\left|\;|\omega_{\text{d}}|\;\right|}\right),$$

$$f_{vlc,r}=k_{v}\left(\nu_{\text{r}}\omega_{\text{d}}-\omega \right),$$

where $\omega_{max}$ defines the maximum angular velocity and ω the current angular velocity of the robot. Please note that the rotation of the robot is only used for reaching a desired orientation and not for avoiding obstacles. This also implies that the additional scaling $k_{vlc}$ is not needed for the calculation of $f_{vlc,r}$ .The total attractive force is a concatenation of the translational and rotational part

$$f_{vlc}=\left[\begin{array}{c} f_{vlc,t}\\ f_{vlc,r} \end{array}\right].$$

#### 2.1.2 Endeffector obstacle avoidance

In this section, we present our adaption of the CF algorithm, which is used in our motion planning framework to efficiently avoid obstacles in the robot’s environment. First, we present the definition and our extensions to nominal boundary following CFs. Then, we introduce the second obstacle avoidance force, which adds a repulsive component. Our proposed algorithm works directly with point-cloud data, thus avoiding computationally intensive and error-prone segmentation of obstacle surfaces. For this purpose, we consider $j=1,\ldots ,n_{o}$ obstacles which are each characterized by a cloud of points xoij∈Oj⊂R3×lj , where $i=1,\ldots ,l_{j}$ . As a result, each point xoij in an obstacle point cloud Oj generates its own magnetic field and its own obstacle avoiding force instead of relying on obstacle surfaces. Moreover, in contrast to a majority of approaches in the literature, we exploit more information about the obstacles beyond the single obstacle point with the minimum distance. This approach reduces sensitivity to sensor noise and improves avoidance behavior in the presence of multiple obstacles, without causing oscillations. The computational load can be managed by reducing the resolution of the sensor point cloud through downsampling. Furthermore, our implementation offers the option to include only the closest $m_{j}\in \left[0,l_{j}\right]$ points of each obstacle for the force calculations. We assume that the obstacle data originates from common motion tracking devices such as laser scanners or camera modules and make the assumption that the point cloud points are reasonably evenly distributed.

##### 2.1.2.1 Nominal circular fields

CFs were first introduced in Singh et al. (1996) and are inspired by the forces acting on a moving charged particle in an electromagnetic field. More specifically, the law of Biot-Savart states that the magnetic field in a distance x from a wire of infinitesimal length dl carrying the current I is defined by

$$dB\left(x\right)=\frac{\mu_{0}}{4\pi }\frac{Idl\times x}{|\;|x|\;{|}^{3}}$$

and will apply the Lorentz force

$$F=q\dot{x}\times B$$

on a particle charged with q and moving with velocity $\dot{x}$ , where $\mu_{0}$ specifies a permeability constant (Halliday et al., 2013). The Lorentz force acts perpendicular to the direction of motion of the charged particle, altering its direction of movement without changing its velocity magnitude.

**FIGURE 1:**
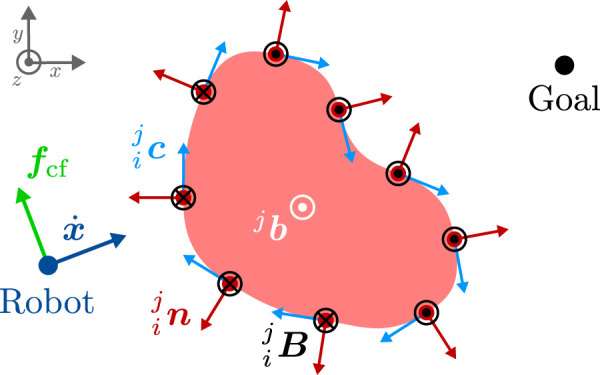
Generation of circular fields (black) and CF force (green) for an obstacle (light red), that is approximated by a point cloud (dark red points) in 2D. In this figure, we use the convention of representing 3D vectors perpendicular to the plane of the diagram with circles: a circle with a dot indicates a vector pointing out of the plane towards the viewer (positive *z*-direction), while a circle with an inscribed cross represents a vector pointing into the plane, away from the viewer (negative *z*-direction). A coordinate system is provided to clarify the orientation of the vectors. In this example, the magnetic field vector (white) is defined as bj=001⊤ and points outside of the page. The current vectors cij are shown in light blue and the surface normals nij in dark red. The resulting circular fields Bij are depicted in black and point either in the positive or negative *z*-direction.

In our application on collision avoidance, we use this physical law as an inspiration and interpret the robot as a charged particle that moves in virtual electromagnetic fields which are generated by virtual currents c on all obstacles points. Figure 1 illustrates the virtual electromagnetic fields generated by a single obstacle in two-dimensional (2D) space and the resulting force on the robot. The direction of the virtual current vector defines the direction of the CF force originating from an electromagnetic field of an obstacle point. As stated in Haddadin et al. (2011), the original definition of the current vector from Singh et al. (1996) is not sufficient because inconsistent orientations of the current vectors on an obstacle lead to oscillations. In order to generate consistent current vectors, we define a magnetic field vector bj∈R3 with ||bj||=1 for each obstacle j , which determines the direction of the current vectors uniformly over the entire obstacle [cf. (Haddadin et al., 2011)].

Note that the magnetic field vector is a crucial element in our obstacle avoidance strategy as it is used to calculate the virtual current vectors, and thus defines the direction in which the robot will pass an obstacle. The choice of the magnetic field vectors therefore has a decisive influence on the overall behavior of the robot and can also be interpreted as the global planning component of our motion planning framework (cf. Section 2.2).

After the definition of the magnetic field vector, the artificial current vector for a point i of an obstacle j can be calculated with

cij=nij×bj,

where nij is the normalized obstacle surface normal pointing outside of the obstacle. Various approaches for surface normal approximation of point clouds exist in the literature. Throughout this paper, we use the functionality provided by the Point Cloud Library (Rusu and Cousins, 2011).

In contrast to previous approaches, our definition of the current vector (12) leads to a continuous current direction over the surfaces of the obstacles (in contrast to (Singh et al., 1996)) and can be easily used to explore multiple trajectories to evade obstacles without depending on the current robot velocity (in contrast to (Ataka et al., 2018b)).

We modify the Biot-Savart law for the use-case of obstacle avoidance, and in our formulation, each point i on an obstacle j generates its own artificial electromagnetic field, that is, its own CF defined as

Bij=cij×d˙ij||d˙ij||.

Here, dij=xoij−x is the distance vector between the robot’s position x and the position of the obstacle point xoij and d˙ij is the respective relative velocity. An example of the electromagnetic fields on a static obstacle is shown in Figure 1.

When the robot moves in such a virtual electromagnetic field, the CF force (a modified version of the Lorentz force) is generated

f^cfij=kcfg1dij+g2dijdij d˙ij||d˙ij||×Bij,

where $k_{cf}>0$ describes a constant gain and dij=||dij||−ds is the distance between an obstacle and the robot including a safety margin $d_{\text{s}}$ . We also use the logistic amplitude functions g1(dij) and g2(dij) defined as

grdij=121+tanhγsl,rγd,r−dij. (2)

**FIGURE 2:**
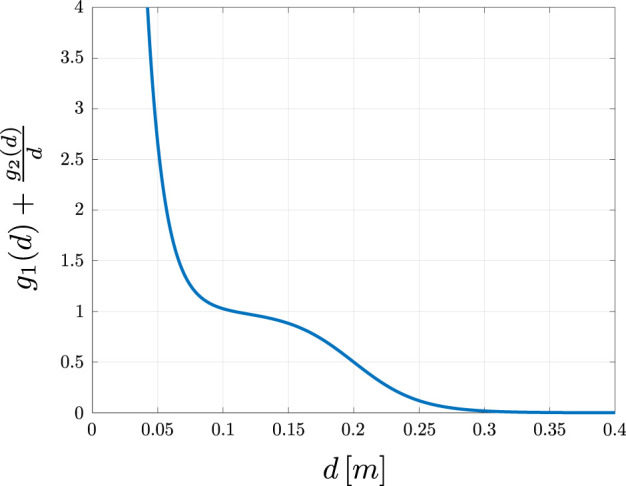
Visualization of the resulting scaling factor g1(dij)+g2(dij)dij using the settings $\gamma_{sl,1}=20.0,\gamma_{d,1}=0.2m,\gamma_{sl,2}=30.0,$ $\gamma_{d,2}=0.01m$ .

Here, the subscript r refers to the parameters associated with the logistic scaling functions $g_{1}$ and $g_{2}$ , which modulate the circular field force magnitude based on the distance between the robot and the obstacle [inspired by Luo et al. (2014)]. These parameters, $\gamma_{sl,r}$ and $\gamma_{d,r}$ control the slope and activation distance of the force, ensuring a smooth activation of the CF force. The subscript r is used to distinguish between different scaling parameters that are applied in the calculation. The effect of the scaling factor g1(dij)+g2(dij)dij is illustrated in Figure 2.This definition of the CF force guides the robot along the boundary of obstacles, preventing collisions. To improve computational efficiency and to mitigate disturbances from obstacle points which are not relevant for the immediate avoidance maneuver, the planner will ignore obstacle points that.

1. Are outside of a range limit around the robot: $|\;|d|\;|\geq d_{max}$ ,
2. Are not directed towards the robot, i.e., the absolute value of the angle between the obstacle surface normal n and the robot-obstacle distance vector d is smaller than 90° : n⋅d≥0 ,
3. The robot moves away from: $n\cdot \frac{\dot{d}}{\left|\;|\dot{d}|\;\right|}\geq \cos\varphi$ and at the same time the relative velocity points towards the goal: $\left(x_{g}-x\right)\cdot \dot{d}>0$ . Here, φ describes the angle between the obstacle surface normal and the relative velocity, and is set in this paper to 85°. This choice ensures that the robot takes into account all obstacle points within a 190° area in front of it, including those to which it moves parallel. The additional condition that the relative velocity points towards the goal is needed to ensure that the robot is able to evade trap-like obstacle shapes.

Using these criteria, the CF force takes the form

fcfij=0,if dij≥dmaxif nij⋅dij≥0if nij⋅d˙ij||d˙ij||≥cosφij∧xg−x⋅d˙ij>0f^cfij,otherwise.

The total CF force from $n_{o}$ obstacles with $m_{j}$ relevant obstacle points (applying the above criteria) then results in

fcf=∑j=0no1mj∑i=0mjfcfij. (3)

Note that the CF force only acts on the translation of the robot, i.e., $f_{cf}\in R^{3}$ . Thus, we append zeros to the force vector for calculating the steering force in Equation 1.

In contrast to other reactive controllers, e.g., the APF approach, our CF planner has multiple advantages. The force is perpendicular to the robot’s velocity, thus it does not dissipate any energy from the system and will not change the velocity magnitude of the robot. Moreover, as shown for point mass robots in Becker et al. (2023b), the planner does not suffer from local minima and consequently will not change the convergence property of attractive fields when no collision with obstacles occurs.

##### 2.1.2.2 Repulsive circular fields

Although the boundary following CF force definition is typically sufficient to prevent collisions, we introduce an additional repulsive force to enable a more robust behavior when the motion of the robot is constrained and the guiding force in Equation 3 might not be sufficient to keep a safe distance to the obstacle. Towards this end, we add an additional repulsive CF-like force, where the artificial current is defined as the negative distance vector

f^cf,repij=−kcf,rep g3dij d˙ij||d˙ij||×dij×d˙ij||dij×d˙ij||, (4)

and we use the scaling factor $k_{cf,rep}\geq 0$ and the definition of g3(dij) from Equation 2. Note that Equation 4 is a simplification of the force from Ataka et al. (2022), which pushes the robot away from the obstacle while maintaining the useful properties of the original CF force, i.e., it is perpendicular to the robot velocity and therefore does not induce local minima.

Additionally, the definition employs similar criteria as the nominal CF force to exclude obstacle points from force generation and superimposes the forces from all obstacle points

fcf,repij=0,if dij≥dmax,repif nij⋅dij≥0if nij⋅d˙ij||d˙ij||≥cosφij∧xg−x⋅d˙>0f^cf,repij,otherwise,fcf,rep=∑j=0no1mj∑i=0mjfcf,repij,

where $d_{max,rep}\leq d_{max}$ is the distance limit for the repulsive CF force.

#### 2.1.3 Attractive force scaling

The combination of the attractive force with obstacle avoidance forces can introduce new problems such as oscillations of the robot, or even induce new local minima and goal convergence issues as discussed in Ataka et al. (2018b). These problems are particularly noticeable in scenarios involving large or non-convex obstacles, where the robot has to move in a direction opposite to the goal position, causing a mutual cancellation of attractive and obstacle avoidance forces. In such cases, a potential solution is to follow the surface of obstacles until they are successfully bypassed, leveraging the boundary-following property inherent in CF forces. Furthermore, in our approach, safety is prioritized by giving precedence to obstacle avoidance over goal convergence. As a result, scaling factors are used exclusively to modify (or potentially deactivate) the attractive force, leaving the CF force unchanged. This strategy ensures that safety concerns are satisfied while addressing the challenges associated with more complex environments, which we show in our goal convergence analysis in a simplified planer setting in Becker et al. (2023b). Therefore, we modify the translational part of the attractive force by introducing the scaling factor $k_{vlc}$

$$f_{vlc}=\left[\begin{array}{c} k_{vlc}f_{vlc,t}\\ f_{vlc,r} \end{array}\right].$$

Please note that the rotation of the robot is only used for reaching a desired orientation and not for avoiding obstacles. This also implies that the additional scaling $k_{vlc}$ is not needed for the calculation of $f_{vlc,r}$ . The goal force scaling factor is defined as

$$k_{vlc}=\left\{\begin{array}{cc} 0\; & \text{if}\;\dot{x}\cdot f_{vlc}\leq 0\wedge |\;|\dot{x}|\;|\leq v_{\min}\wedge |\;|x_{g}-x|\;|>\xi \\ w\; & \text{otherwise} \end{array}\right.$$ (5)

with $w=w_{1}w_{2}w_{3}$ and $w_{1},w_{2},w_{3}\geq 0$ .

Note that the CF force does not change the magnitude of the robot velocity (cf. Becker et al., 2023b), which is therefore only modified by the VLC. Using Equation 5 and therefore deactivating $f_{vlc}$ when it works against the current motion direction while the velocity is below or equal to a defined $v_{\min}$ , we ensure that the robot will only decrease its velocity below this minimum, when the robot is in the vicinity ξ>0 of the goal pose.

Consequently, when the attractive goal force is deactivated, the robot does not stop. Instead, the CF forces guide the robot to follow the obstacle’s boundary, ensuring smooth obstacle avoidance without halting the motion towards the goal. The first two factors $w_{1}$ and $w_{2}$ are taken from Ataka et al. (2018b). The first factor is used to limit the VLC force when the robot is close to obstacles

$$w_{1}=1-\exp^{-\frac{\left|\;|d|\;\right|}{\gamma_{o}d_{max}}},$$

where $\gamma_{o}>0$ is a constant scaling factor and $\left|\;|d|\;|=|\;|x_{o}-x|\;\right|$ is the minimal distance between the robot end effector x and the closest obstacle point $x_{o}$ . When the robot approaches an obstacle, the VLC force converges to zero, prioritizing collision avoidance over goal convergence.

The second term reduces the attractive force when an obstacle is between the robot and the goal position and increases the force otherwise

$$w_{2}=1-\frac{\left(x_{g}-x\right)\cdot d}{\left|\;|x_{g}-x|\;||\;|d|\;\right|}.$$

This factor is zero when the goal vector $x_{g}-x$ and the distance vector d point in the same direction, it is equal to one if the two vectors are orthogonal to each other and it doubles the influence of the VLC force when the vectors point in opposite directions.

The factor $w_{3}$ is introduced to handle environments with non-convex obstacles, where obstacle configurations might cause opposing VLC and CF forces. This would again result in a decrease of the robot’s velocity as the goal force acts against the robot’s direction of motion. In such a case the CF force should be dominating to guide the robot along the obstacles’ boundaries until the obstacle is passed. This can be achieved by scaling the VLC force with the scalar product of the normalized VLC force and the current robot velocity in the form

$$w_{3}=\left\{\begin{array}{cc} 1+\frac{\dot{x}\cdot f_{vlc}}{\left|\;|\dot{x}|\;||\;|f_{vlc}|\;\right|}\; & \text{if }\dot{x}\cdot f_{vlc}<0\\ 1\; & \text{otherwise.} \end{array}\right.$$

#### 2.1.4 Robot body obstacle avoidance

**FIGURE 3:**
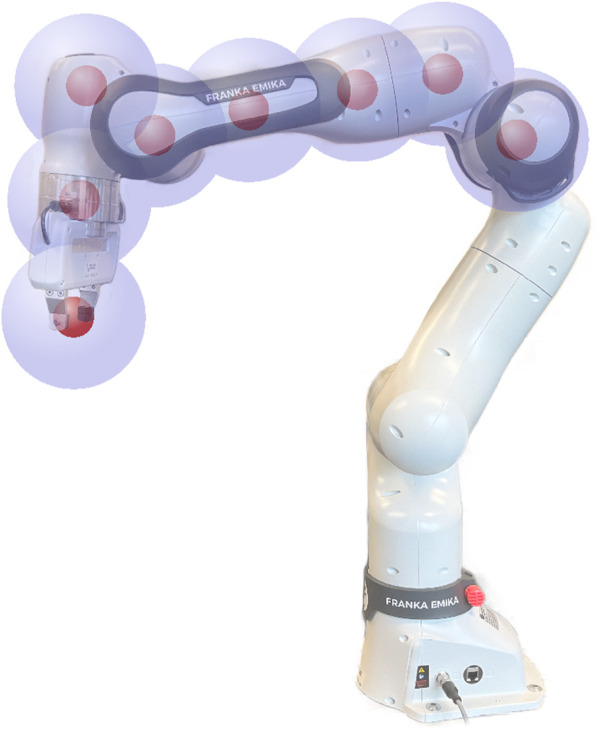
Visualization of possible robot control points including a spherical approximation of the robot.

To enable obstacle avoidance for the entire robot body, we define $n_{\text{cp}}$ additional control points along its structure, as exemplarily shown for a 7-DoF robot arm in Figure 3. The control points should be placed on prominent points of the robot so that the whole structure can be moved away from obstacles. To ensure that the robot structure keeps a safe distance to obstacles, the distance to the control points is also calculated using a safety margin, resulting in a spherical approximation of the robot arm. Note that for the example in Figure 3, we have refrained from adding more control points on the lower links as their obstacle avoidance capabilities are limited. We assume that the robot needs to reach a Cartesian goal pose and that we do not have any information about possible final joint configurations. Consequently, we do not apply an attractive goal force on the control points and the force on a control point $k\in \left[1,n_{cp}\right]$ only consists of the respective CF-based obstacle avoidance forces

fcpk=fcfk+fcf,repk.

#### 2.1.5 Joint limit avoidance

To ensure safe operation of robotic manipulators, it is crucial to consider their physical constraints, specifically their joint limits. We use sigmoidal scaling functions as defined in Equation 2 to construct a repulsive vector that pushes the joints towards their center position. The repulsive vector consists of two parts. The first element pushes the joints towards their center with a comparatively small magnitude to keep the robot in configurations that allow a wide range of motions. The second component is activated when a joint is close to its limits and generates repulsive vectors with greater magnitude to move the robot away from its limits. Towards this end we first normalize each joint $p\in \left[1,n_{dof}\right]$ to the range qnp∈[−1.0,1.0] using

qnp=−1+2qp−qminpqmaxp−qminp.

**FIGURE 4:**
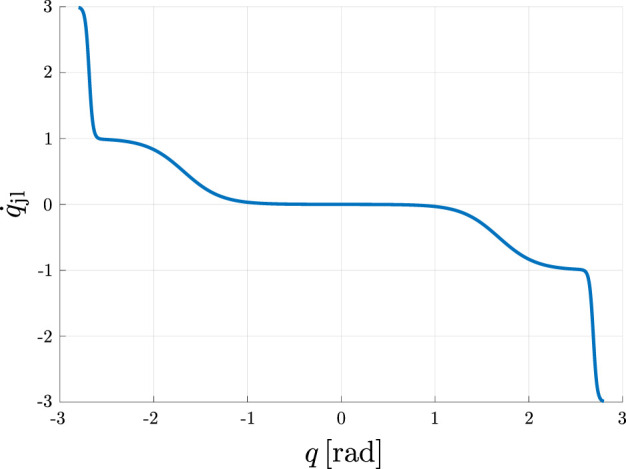
Visualization of the repulsive vector ${\dot{q}}_{\text{jl}}$ for a joint with the limits $q_{\text{max}}=2.8\;rad$ and $q_{\text{min}}=-2.8\;rad$ using the settings $k_{jc}=1.0,k_{jl}=2.0,\gamma_{sl,jc}$ $=7.0,\gamma_{d,jc}=0.4,\gamma_{sl,jl}=70.0,\gamma_{d,jl}=0.04$ .

Here, qminp and qmaxp are the minimum and maximum position limits of a joint p . The repulsive vector is then calculated using each joint individually

q˙jlp=kjc gjcqnp+kjl gjlqnpif qnp<0−kjc gjcqnp−kjl gjlqnpif qnp>00otherwise,

where $k_{jc},k_{jl}\geq 0$ are scaling factors of the repulsive vector for the joint centering and the joint limit avoidance components, respectively and gjc(qnp),gjl(qnp) are sigmoidal functions as defined in Equation 2. An illustrative example for the magnitude of this repulsive vector for a single joint is shown in Figure 4.

#### 2.1.6 Manipulability

The manipulability of a robotic manipulator is a useful criterion for quantifying the influence of joint movements on the end effector, thus providing a measure how easy or difficult it is to change the robots pose in its Cartesian operational workspace. A high manipulability indicates that only small movements of the joints are necessary to result in a large motion of the EE while the manipulability converges to zero when the robot is close to a singularity. Thus, it also provides information about the relative distance to singular configurations. The manipulability index, introduced in Yoshikawa (1985), is given by

$$\mu \left(q\right)=\sqrt{det\left(J_{ee}\left(q\right)J_{ee}{\left(q\right)}^{⊤}\right)}.$$

The gradient of the manipulability can be used to calculate joint velocities that maximize the manipulability as presented in Dufour and Suleiman (2020)

$${\dot{q}}_{\text{m}}=k_{m}{\left(\frac{\partial \mu \left(q\right)}{\partial q}\right)}^{⊤},$$

where $k_{m}>0$ is a constant gain.

#### 2.1.7 Self-collision avoidance

To avoid self-collisions, we approximate the structure of the robot by spherical obstacles with a radius $d_{\text{sc}}$ and use repulsive forces similar to Luo et al. (2014)

fsclk=−ksc gscdlk dsclk||dsclk||if dlk<dmax,sc0otherwise, (6)

where dsclk=xrsl−xcpk defines the distance vector between a control point k and a repulsive sphere l , gscdlk is a sigmoidal function as defined in Equation 2, and $k_{sc}>0$ is a scaling factor. The force is applied only if the distance dlk=||dsclk||−dsc is less than a threshold $d_{max,sc}$ . Similar to the CF forces, the repulsive forces of all $n_{\text{sc}}$ self-collision obstacles are superposed, leading to the following self-collision avoidance force on a control point $k\in \left[0,n_{cp}\right]$

fsck=∑l=0nscfsclk,

where the EE is considered as the control point with k=0 . When the self-collision avoidance force is used, the EE steering force and the forces on the control points are updated to

fsee=fvlc+fcf+fcf,rep+fsc0,fcpk=fcfk+fcf,repk+fsck.

Note that in practical implementations, many of these repulsive forces may not be necessary or may even lead to infeasible or detrimental forces, e.g., if a control point is located on the same link as a repulsive sphere. Therefore special care has to be taken when defining the repulsive spheres on the robot structure. In our implementations, it was sufficient to place a single virtual repulsive sphere in the base of the robot which generates a force only on the EE.

#### 2.1.8 Joint velocity damping

We introduce an additional joint velocity component that has a damping effect using

$${\dot{q}}_{\text{damp}}=-k_{damp}\dot{q},$$

with the scaling factor $k_{damp}\geq 0$ . Adding ${\dot{q}}_{\text{damp}}$ , which opposes the current velocity direction, to the total velocity control command resulting from the motion planning strategy, yields a component of the tracking controller output that has a damping effect and prevents undamped motions in the nullspace.

#### 2.1.9 Robot control signal

In this section, we describe how we transform the steering forces, joint velocity commands and repulsive vectors into feasible reference signals for joint space control.

Consider the well-known equations of motion for a robotic manipulator

$$\tau_{\text{ee}}+J_{\text{ext}}^{⊤}f_{\text{ext}}=J_{ee}^{⊤}\left(q\right)\left(M_{\text{c}}\left(q\right)\ddot{x}+c_{\text{c}}\left(q,\dot{q}\right)+g\left(q\right)\right),$$ (7)

where $M_{\text{c}}\left(q\right)$ is the Cartesian inertia matrix, $c_{\text{c}}\left(q,\dot{q}\right)$ describes the Coriolis and centrifugal terms, g(q) are the gravitational forces, $J_{ee}\in R^{6\times n_{dof}}$ is the Jacobian, describing the relation between the velocities of the $n_{dof}$ joints and the EE velocity and $f_{\text{ext}}$ are external forces acting on the robot, where $J_{\text{ext}}$ is the Jacobian of the location where the external forces apply. This formulation enables direct torque control of the robotic manipulator and accommodates additional external forces commonly encountered during human-robot interaction, as discussed in Haddadin et al. (2010).

However, this paper specifically focuses on collision avoidance and we employ a separate joint space tracking controller for robot control. We assume that this joint space controller sufficiently compensates for gravity and dynamics. Consequently, we only need to transform the virtual task space steering forces generated by the proposed motion planning framework into desired joint reference signals. Furthermore, recall that we do not consider real forces; instead we interpret the artificial steering force as the desired EE acceleration, which allows us to ignore inertial forces. Thus, we use $f_{s_{ee}}=\ddot{x}$ , set the inertia matrix in joint space to the identity matrix M(q)=I and disregard the gravitational and Coriolis terms in Equation 7. This implies ${\ddot{q}}_{\text{ee}}=\tau_{\text{ee}}$ and leads to the following definition of the Cartesian inertia matrix^2^

$$M_{\text{c}}\left(q\right)={\left(J_{ee}\left(q\right)M{\left(q\right)}^{-1}J_{ee}^{⊤}\left(q\right)\right)}^{-1}$$

$$={\left(J_{ee}\left(q\right)IJ_{ee}^{⊤}\left(q\right)\right)}^{-1}.$$

This approach simplifies Equation 7, allowing us to eliminate the inertia matrix from our calculations. This speeds up the planning process and avoids known issues associated with modeling inaccuracies of the inertia matrix (cf. Nakanishi et al., 2008).

Inserting $M_{\text{c}}\left(q\right)$ into Equation 7 and neglecting the gravitation and dynamic components yields

$${\ddot{q}}_{\text{ee}}=J_{ee}^{\#}\left(q\right)f_{s_{ee}},$$

where $J_{ee}^{\#}=J_{ee}^{⊤}{\left(J_{ee}J_{ee}^{⊤}\right)}^{-1}$ is the Moore-Penrose pseudo inverse. Note that we additionally limit each joint individually to its maximum acceleration.

Ideally, the forces on the control points should be transformed into the nullspace of the main task, i.e., the control of the EE pose. However, our experiments indicate that consistently achieving full-body obstacle avoidance without interfering with the primary EE task is challenging. Avoiding obstacles within the null space of the EE task is only feasible in few scenarios where simple obstacle avoidance movements are sufficient. Thus, the forces on the control points are considered as virtual external forces on the robot body. These forces are then transformed to joint accelerations using

q¨cpk=Jcpk⊤q fcpk,

where Jcpk is the Jacobian of the position of the k th control point. The total joint acceleration command is calculated by superimposing the desired accelerations from all control points

q¨cmd=q¨ee+∑k=0ncpq¨cpk.

This approach ensures an indirect weighting of the joint accelerations based on the magnitude of the respective task space force. The influence of the control point acceleration on the total robot motion increases as the control point gets closer to an obstacle. Additionally, we employ a safety fallback strategy when the distance between a control point and an obstacle exceeds a lower threshold (cf. Section 2.2.4). Note that the simplification described above and the direct application of the forces on the control points affect the ability of the robot to follow the desired EE trajectory exactly. However, the proposed approach leads to efficient convergence to task space goal poses while avoiding obstacles, even in dynamic and complex settings, as demonstrated in Section 3.

We consider obstacle avoidance as the primary objective and the supplementary measures for joint centering, manipulability and damping as less important. Thus, these desired joint velocities and repulsive vectors are projected into the nullspace of the EE using the method described in (Siciliano and Khatib, 2008).

Finally, we calculate the reference signals for the next sampling step of the joint space controller. We integrate the acceleration control reference for generating appropriate inputs to a desired joint controller.

$${\dot{q}}_{\text{cmd}}=\dot{q}+{\ddot{q}}_{\text{cmd}}T_{c}+\left(I-J_{ee}^{\#}\left(q\right)J_{ee}^{⊤}\left(q\right)\right)\left({\dot{q}}_{\text{m}}+{\dot{q}}_{\text{jl}}+{\dot{q}}_{\text{damp}}\right),$$ (8)

$$q_{\text{cmd}}=q+{\dot{q}}_{\text{cmd}}T_{c},$$

where $T_{c}$ is the control step time. Similarly to the joint acceleration, each joint is limited individually to its maximum velocities and angle. In our applications, we use a joint impedance controller, which considers joint velocity and joint angle reference signals as inputs.

### 2.2 Unifying global planning and reactive control

Although our reactive CF-based control component performs well in a local scope of the environment, it faces inherent limitations typically associated with locally reactive controllers. These limitations have been extensively discussed in the related work (cf. Section 1.2) and primarily stem from the omission of global environmental information and constrained exploration capabilities. Traditional CF approaches, which function solely as local planners, perform suboptimally when attempting to find global solutions, resulting in trajectories that are generally less efficient. Moreover, despite the inherent absence of local minima in CFs, it is still possible to design trap scenarios that restrict the robot’s movement. Note that this is not equivalent to the local minima encountered in APF approaches, as the robot does not come to a standstill but instead becomes trapped in limit cycles.

In (Becker et al., 2021) we developed the CFP planner, which uses a predictive virtual agent framework to efficiently explore the global environment by simulating the robot using different settings for the magnetic field vector. However, in contrast to our previous work, the extension to robotic manipulators using additional control points on the robot leads to a significant increase of virtual agents, especially in the case of many obstacles. Furthermore, the framework is not able to determine which avoidance directions around obstacles of the different control points are not compatible with each other before simulating the whole robot motion. Thus, many combinations of magnetic field vectors for the different control points will lead to infeasible trajectories or even to collisions. Consequently, a considerable amount of computing power is wasted by simulating these magnetic field vector sets.

To compensate for these disadvantages, we introduce the concept of ICF, which extends the capabilities of the local CF approach by incorporating and extracting global environmental information. The method is not intended to operate as a standalone global planner; instead, it is tightly integrated into the CF planning component. The integration aims to unify global planning and reactive control, bridging the gap between global environment exploration and reactive collision avoidance. The heavily parallelized design enables instantaneous responses to dynamic obstacle motion, and unpredictable changes in partially unknown environments, even when the global component faces challenges in finding a solution.

The general idea of ICF is to leverage the strengths of global and local motion planning strategies by extracting useful information from a global pre-planner. This information is then transferred to and evaluated by an adapted virtual agents framework to improve reactive motion generation. Throughout the development, a significant emphasis has been placed on maintaining the reactivity of the CF planner, even when the global planning component fails to find a solution.

The process of generating a control reference signal within our ICF framework is divided into four distinct phases.

1. Initially, we employ a global configuration space motion planner to create a (coarse) joint trajectory (cf. Section 2.2.1).
2. Following this, we extract global information from this coarse trajectory in the form of magnetic field vectors for all control points and obstacles, which serves as a basis for reactive CF-based motion planning (cf. Section 2.2.2). It is important to note that when referring to control points, the EE is included unless otherwise specified.
3. Subsequently, multiple predictive agents are created using the extracted magnetic field vectors to process and evaluate the global motion plan (cf. Section 2.2.3).
4. The parameters of the best agent are then transferred to the real robot and used to generate the reactive joint space commands (as described in Section 2.1.9).

These different phases are executed in parallel, each with its respective sampling rates.

#### 2.2.1 Global trajectory generation

The main purpose of the global pre-planner is to infer estimates for feasible global trajectories from the current robot pose to a Cartesian goal pose in the configuration space. In particular, finding non-conflicting avoidance directions around the obstacles for all control points is more important than generating short and smooth paths. Consequently, we use a rather coarse discretization of the global planner for shorter planning cycles $T_{global}$ . The global planner is continuously running and restarted after $T_{global}$ seconds with updated obstacle and robot information. When a successful trajectory is found during the planning time, the trajectory is passed to the global information extraction module.

Due to frequently proven successful results, short planning times, and wide availability, we use sampling-based planners for the global trajectory generation. In particular, we use the MoveIt! motion planning framework because it features a variety of sampling-based planners and supports point clouds as a format for obstacle representation (Coleman et al., 2014). However, other configuration space planners can also be used and exchanged easily. Note that the global motion planning is done in a static snapshot of the environment while the actual reactive creation of the control reference (cf. Section 2.1.9) is always done with the most current environment information.

#### 2.2.2 Global information extraction

**FIGURE 5:**
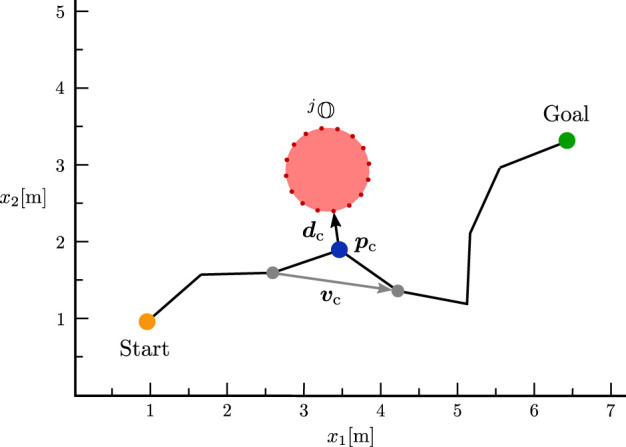
Visualization of Algorithm 1 in a simplified 2D representation of the environment.

**FIGURE 6:**
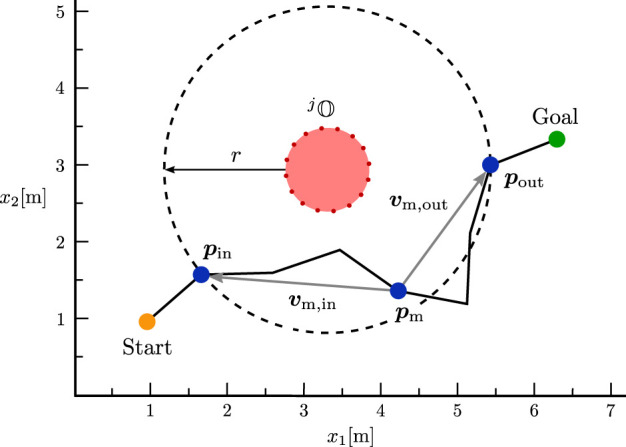
Visualization of Algorithm 2 in a simplified 2D representation of the environment.

Whenever the global pre-planner finds a successful joint trajectory, the global information extraction module is triggered. The joint trajectory is then transformed into separate Cartesian trajectories for each control point using forward kinematics

xcpkt=Tcpkqt0001⊤,

where Tcpk is the Denavit-Hartenberg (DH) matrix that defines the transformation from the base frame to the control point k . The avoidance direction of each control point around each obstacle is then extracted from the Cartesian trajectories and the corresponding magnetic field vectors are calculated using both of the following algorithms. Visualizations of both algorithms are shown in Figures 5, 6 in 2D example setups.For each control point k and for each obstacle j described by the points xoij∈Oj⊂R3×mj , the following calculations are performed.

Algorithm 1 First option for extracting the avoidance direction around an obstacle from a pre-planned path. This algorithm captures the avoidance motion at the closest obstacle point and is particularly effective when the obstacle is being avoided in a uniform motion without directional changes.

1. Find the closest point $p_{\text{c}}$ of the trajectory Xcpk=xcpk(t)∣t∈[0,Tglobal] to the obstacle j
pc=xcpkτwithτ=argmint∈0,Tglobalminxoij∈Oj||xcpkt−xoij||.
2. Calculate the (approximate) direction of movement $v_{\text{c}}$ at the closest point $p_{\text{c}}$
vc=xcpkτ+1−xcpkτ−1.
3. Calculate the vector $d_{\text{c}}$ pointing from $p_{\text{c}}$ to the closest obstacle point xocj
dc=xocj−pcwithxocj=argminxoij∈Oj||xoij−pc||.
4. Define the magnetic field vector bj as
bj=dc×vc||dc×vc||.

Algorithm 2 Second option for extracting the avoidance direction around an obstacle from a pre-planned path. This algorithm generalises the avoidance motion in an environment around the obstacle in order to better map the resulting total evasive direction and to ignore additional movements and reconfigurations that do not contribute to the avoidance direction.

1. Find the first point $p_{in}$ of the trajectory xcpk in a ball of a predefined radius r around the obstacle j
pin=xcpkτminwithτmin=mint∈0,Tglobal,xoij∈Ojt∣||xcpkt−xoij||≤r.
2. Find the last point $p_{out}$ of the trajectory xcpk in a ball of a predefined radius r around the obstacle j :
pout=xcpkτmaxwithτmax=maxt∈0,Tglobalminxoij∈Ojt∣||xcpkt−xoij||≤r.
3. Find the point pm=xcpk(τ) with τ being the largest integer less than or equal to the midpoint in $\left[\tau_{\min},\tau_{\max}\right]$ , i.e.,
$$\tau =\left\lfloor 0.5\left(\tau_{\max}+\tau_{\min}\right)\right\rfloor .$$
4. Calculate the vectors $v_{m,in}$ pointing from $p_{m}$ to $p_{in}$ and $v_{m,out}$ from $p_{m}$ to $p_{out}$
$$\begin{array}{cc} v_{m,in} & =p_{in}-p_{m},\\ v_{m,out} & =p_{out}-p_{m} \end{array}$$
5. bj is defined perpendicular to the plane through the three points $p_{in}$ , $p_{out}$ and $p_{m}$
bj=vm,in×vm,out||vm,in×vm,out||.

The quality of the resulting avoidance motion from both methods highly depends on the environment and the global trajectory. The first algorithm captures the avoidance motion only at the closest obstacle point. Therefore, it only accurately represents the avoidance movement if the obstacle is avoided through a uniform motion without any directional changes. In contrast, the second algorithm defines a surrounding area around the obstacle to recreate a resulting homogeneous avoidance direction around that region. As a result, any additional movements or changes in direction within this area are neglected, as they do not contribute to the avoidance maneuver. However, in some cases, it may be necessary to perform additional motions close to an obstacle. The relevance of these motions always depends on the specific scenario, e.g., additional motions may be necessary to reconfigure the robot joints and reach the goal without hitting the joint limits. Therefore, we use both methods to calculate magnetic field vectors, which are adopted by the predictive agents as described in the next section. Nevertheless, we simplify a potentially complex avoidance motion to a single vector per obstacle and control point. Thus, the resulting motion of the ICF planner is expected to deviate from the global trajectory. However, we would like to emphasize again that we only use the global planner to estimate feasible, non-contradictory avoidance directions, which are subsequently simulated and evaluated before being used to enable reactive control of the real robot.

#### 2.2.3 Adapting the virtual agent framework

After transforming the trajectories of the global pre-planners into magnetic field vector sets, we utilize the virtual agent framework described in Becker et al. (2021) to process this global information. The framework enables us to identify the influence of selected control parameters, such as the magnetic field vectors, on the robot in a current perception of the environment under simplified dynamics. Within the framework, the robot is represented in this environment snapshot by virtual predictive agents, each with a specific set of parameters P . In order to adequately account for the new information from the global pre-planner, the process for creating and deleting predictive agents from Becker et al. (2021) is redesigned as described in the following.

##### 2.2.3.1 Virtual agent creation

Whenever the global pre-planner and information extraction module generate new magnetic field vectors, new virtual agents using these magnetic field vectors are created. Moreover, if the maximum number of predictive agents $n_{a,max}$ has not been reached, additional agents with different parameter sets P are created. The creation of new predictive agents follows a specific order upon receiving new magnetic field vectors.

1. Create one agent with the current best parameter set $P_{\text{best}}$ and the current best magnetic field vectors $B_{\text{best}}$ .
2. Create one agent with the current best parameter set $P_{\text{best}}$ but with the new magnetic field vector set $B_{\text{A}1}$ from Algorithm 1.
3. Create one agent with the current best parameter set $P_{\text{best}}$ but with the new magnetic field vector set $B_{\text{A}2}$ from Algorithm 2.
4. Create additional agents with a parameter set that differs in an arbitrary parameter $m_{1}$ from $P_{\text{best}}$ if the number of agents is less than $n_{a,max}$ .
5. Create additional agents with a parameter set that differs in parameter $m_{1}$ from $P_{\text{best}}$ and uses $B_{\text{A}1}$ if the number of agents is less than $n_{a,max}$ .
6. Create additional agents with a parameter set that differs in parameter $m_{1}$ from $P_{\text{best}}$ and uses $B_{\text{A}2}$ if the number of agents is less than $n_{a,max}$ .
7. Repeat steps 4-6 with other parameters $m_{p}$ or with another feasible option for the parameter $m_{1}$ until a total of $n_{a,max}$ agents have been created.

In this paper, we use the virtual agent framework primarily for the magnetic field vector $b\in B\subset R^{n_{\text{cp}}\times n_{\text{o}}}$ of the $n_{\text{cp}}$ control points (including the EE). However, as described previously other parameter choices can also be simulated. In our approach, the supplementary forces described in Sections 2.1.5, 2.1.6 and 2.1.8 are defined as optional parameters for calculating the final control command [cf. Equation 8]. Consequently, we add binary activation flags for all supplementary forces to the parameter vector P . This enables the deactivation of supplementary forces as needed, allowing for only necessary calculations to be performed at any given time. For instance, we define $m_{1}$ to be the activation flag of the manipulability gradient calculation method from Section 2.1.6. As a result, instead of including the manipulability velocity vector in the reference command calculation Equation 8 in every simulation, we simulate both a set of agents that applies the manipulability gradient to the resulting velocity command and a set of agents without it. This procedure ensures that the command for the real robot is calculated using the optimal set of forces and prevents situations, where, e.g., the manipulability gradient might interfere with the force on a control point that is close to an obstacle.

##### 2.2.3.2 Virtual agent simulation

The virtual agents are then simulated using the control commands from Section 2.1.9, assuming simplified dynamics and that the controller follows the joint commands perfectly, i.e.,

$$\begin{array}{ll} q\left(t+1\right) & =q\left(t\right)+{\dot{q}}_{\text{cmd}}\left(t\right)\Delta T\\ \dot{q}\left(t+1\right) & ={\dot{q}}_{\text{cmd}}\left(t\right)\\ {\dot{x}}_{\text{ee}}\left(t+1\right) & =J_{\text{ee}}\left(q\left(t+1\right)\right)\dot{q}\left(t+1\right). \end{array}$$

This procedure is repeated until the simulated agent reaches the goal pose $x=x_{g}$ . However, virtual agents are only simulated for a defined maximum number of prediction steps $n_{ps}$ at a time, after which the framework proceeds with the simulation of a different agent. It is essential to note that parallel computation of multiple agents remains possible and is actively exploited. This method was introduced to ensure that all agents are treated with the same priority and progress through the simulation at a similar rate. In practical implementation on conventional computing devices, it is otherwise difficult to ensure a fair distribution of computing power over several threads. This is particularly important when more agents were created than the computation device can handle concurrently. Specifically, this precautionary measure prevents agents, which might never reach the goal or follow long suboptimal trajectories (e.g., due to poor choices of magnetic field vectors), from indefinitely blocking the available computation slots. During the simulation it is possible to also simulate the motion of dynamic obstacles, for which we use a constant velocity model in our simulations.

##### 2.2.3.3 Virtual agent evaluation

In contrast to Becker et al. (2021), the evaluation of predictive agents is performed asynchronously instead of evaluating all agents simultaneously at a fixed time interval. Each agent is evaluated and compared to the current best agent immediately after its simulation, which is interrupted either after $n_{ps}$ steps or upon reaching the goal. If an agent has a higher reward, it is validated whether the position of the predicted trajectory of the new parameters for the latest time step approximately matches the latest real agent position. Discrepancies may arise when the robot took a different avoidance direction around an obstacle, dynamic obstacles influenced the robot trajectory, or due to discretization errors. In order to mitigate the influence of discretization errors, the threshold for detecting deviations should be adjusted. Nonetheless, with time, the discretization error is expected to surpass the threshold, indicating a potentially outdated predicted trajectory. In all cases, the respective agent is deleted and the parameter set is not updated. Otherwise, the real robot uses the new best parameter set.

We use the following criteria, prioritized by the reward gains $\varrho_{g}\geq \varrho_{d}\geq \varrho_{tl}\geq \varrho_{jl}\geq \varrho_{s}\geq \varrho_{o}\geq \varrho_{mfv}\geq 0$ for evaluating a virtual agent.

1. Reaching the goal with the EE without colliding is typically a complex task, and thus an agent p that approaches a distance $d_{gd}$ around the goal gets a high reward $\varrho_{g}$ . Otherwise, only a smaller reward $\varrho_{d}$ is granted that decreases exponentially with a higher remaining distance to the goal

rgdp=ϱgif ||xg−xeepNpsp||≤dgd,ϱde−||xg−xeepNpsp||γgdotherwise,

where $\gamma_{gd}>0$ is a constant scaling factor and Npsp is the total number of steps that agent p has been predicted. Appropriate scaling of $\varrho_{g}$ ensures that an agent that reached the goal without collision will always receive a higher total reward than an agent who has not yet done so.

2. The distance covered by the EE of a manipulator is often a less meaningful evaluation criterion than the path length traveled by a mobile robot. Instead, we use the duration of the current prediction as a criterion for rewarding shorter motions

rtlp=ϱtl−NpspTs.

3. We favour trajectories that result in a lower percentage of joint limit avoidance forces

rjlp=ϱjl1−∑n=0Nps−1p||q¨jlpn||||q¨eepn+q¨cppn+q¨jlpn||.

4. We also use the manipulability index to avoid singularities by rewarding a higher minimum manipulability of the agent trajectory

rsp=ϱsminn∈0,Npspμqpn.

5. We reward agents, which pass obstacles with greater clearance by including the minimal distance of all control points and the EE to all obstacles defined as

dminp=minn∈0,Nps,k∈0,ncp,xoijn∈Ojn,j∈1,no||xcppkn−xoijn||,

where k=0 again denotes the EE. The resulting reward is then given by

rop=ϱo1−edmin−dminp.

6. To prevent abrupt changes in force direction or oscillations of the robot, we aim to avoid constant switches between agents with similar scores. This is achieved by rewarding agents that have similar avoidance directions as the current best agent. We compare the magnetic field vectors of all control points and obstacles to determine the similarity

rmfvp=ϱmfv1−1noncp∑j=0no∑k=0ncp||bkjbest−bkj||.

The total reward is defined as the sum of all individual rewards

rtp=rgdp+rtlp+rjlp+rsp+rop+rmfvp.

It is important to note that we do not pass the calculated trajectory to the real robot. Instead, the real robot adapts the parameters of the best agent and calculates its control command in parallel to the simulation of the virtual agents and to the planning of the global pre-planner. This procedure ensures reactive behavior of the overall planning framework and also allows that the simulation of the virtual agents are performed with a coarser step time to speed up the global computation process.

##### 2.2.3.4 Virtual agent validation

**FIGURE 7:**
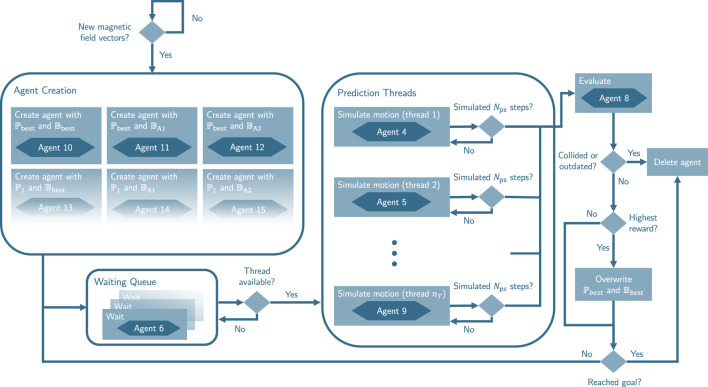
Activity diagram of adapted virtual agent framework from ICF approach.

In environments with dynamic obstacles, it is natural for the actual system behavior to differ over time from the prediction due to the asynchronous simulation and the different time step discretization. Our approach involves regular validations to ensure that the predicted trajectory of the current best agent remains consistent with the actual robot trajectory and avoids collisions with obstacles, for instance, from motions of dynamic obstacles. If deviations occur, the predicted trajectory is invalidated, and the best agent reward is reset to zero. After validation, a new agent is created with the current robot parameter set and state. This ensures continuous simulation of the best agent and maintains comparability of its reward with new agents. The planner also initiates re-planning when all agents have either reached the goal or have been deleted. This process also involves creating a predictive agent at the current robot state using the best parameter set. Setting the reward to zero may result in frequent changes of the best agent and could lead to undesired behavior, such as oscillations. To mitigate this, the comparison of rewards can be delayed until a minimum number of agents $n_{a,min}$ were evaluated. During this transition period, the real robot continues moving using the previous best parameters and using the safety-improving fallback, which is described in the following section. The whole process is depicted in Figure 7.

#### 2.2.4 Safety-improving fallback

During motion planning, it may happen that none of the predicted trajectories match the current state of the environment, either because of unpredictable movements of the obstacles or because the robot cannot follow the predicted trajectories with sufficient accuracy. Additionally, this could lead to close distances between a control point and obstacles. In both cases, collision avoidance has absolute priority and the robot switches to a safety-improving fallback behavior. Here, the CF forces on the control points on the robot structure are replaced by repulsive forces that are defined similarly to the self-collision avoidance force in Equation 6. The force scaling from the sigmoidal function (cf. Equation 2) ensures that only obstacle points within a limited range around the robot generate a force. The maximum range can be adjusted by the factor $\gamma_{d}$ , while $\gamma_{sl}$ determines the rate at which its level of influence increases. The total force from all obstacle points on a control point is defined by

frpfk=∑j=0no∑i=0mjfrpfikj.

Moreover, we change the nullspace of the supplementary forces, i. e., the manipulability gradient, the joint limit avoidance and the damping velocity, whenever a control point is close to an obstacle. Instead of projecting the forces in the nullspace of the EE pose, the forces are projected into the augmented nullspace of the EE translation and the translation of the closest control point. Accordingly, the steering forces and the joint velocity control reference are calculated with.

fsee=fvlc+frpf0,

fcpk=frpfk

$${\dot{q}}_{\text{cmd}}=\dot{q}+{\ddot{q}}_{\text{cmd}}T_{c}+\left(I-J_{safe}^{\#}\left(q\right)J_{safe}^{⊤}\left(q\right)\right)\left({\dot{q}}_{\text{m}}+{\dot{q}}_{\text{jl}}+{\dot{q}}_{\text{damp}}\right),$$

where Jsafe⊤=Jee,t⊤Jcp,tc⊤ is defined by the Jacobian of the EE position $J_{ee,t}\in R^{n_{dof}\times 3}$ and the Jacobian of the position of the control point that has the minimum distance to the obstacles Jcp,tc∈Rndof×3 . Note that this definition of the repulsive force inherits the same disadvantages as APF, notably the introduction of local minima. However, we want to highlight again, that this is a rare occurrence where collision avoidance takes priority over goal convergence. Furthermore, it should be noted that the safety improvement fallback mechanism is disabled once an agent discovers a feasible path to the goal pose.

## 3 Simulations and experiments

In this section, we evaluate the performance of the ICF planner across various static and dynamic environments. Given that the ICF planning framework can be used in combination with arbitrary global motion planning approaches, we first conduct a comparative analysis to determine the most suitable pre-planner within our proposed framework (cf. Section 3.1). Subsequently, in Section 3.2, we compare the ICF planner in combination with the two most effective pre-planners against other state-of-the-art motion planners, encompassing sampling-based, optimization-based, and reactive planning approaches.

Finally, we demonstrate the efficacy of the motion planning framework in a dynamic real-world scenario, where a Franka Emika Research 3 robot has to reactively avoid colliding with a human in its workspace (cf. Section 3.3).

The simulative evaluations are conducted in a kinematic simulation environment without disturbances, wherein the current positions and velocities of the obstacles are known, but their future behaviour is not. We use a C++ implementation on a computer equipped with an AMD® Ryzen 9 5950X CPU with 16 cores, operating at 3.4 GHz and an NVIDIA GeForce RTX 2070 Super GPU.

The same computer is used for calculating the planning commands in the real world experiment. These commands are send to an Intel® Core i7-6700 CPU with 4 cores, 3.4 GHz running a real-time kernel, which is necessary for controlling the Franka Emika robot. For detecting and tracking the human, we use a Sterolabs ZED 2 camera and the corresponding ZED ROS wrapper^3^ on a third computer with an Intel® Core i7-6700 CPU with 16 cores, 3.4 GHz and an NVIDIA GeForce RTX 4060 GPU.

Our simulations and experiments involve complex environments with multiple dynamic obstacles. Thus, we uploaded videos of the execution of all scenarios in our accompanying repository Becker et al. (2024). The repository includes comprehensive listings of the parameters utilized in all simulations and experiments.

### 3.1 Performance of different global pre-planners

First, we evaluate the performance of the ICF planner using different global pre-planners. Towards this end, we employed 20 different sampling-based planners from the MoveIt! framework, executing them in five static and five dynamic environments. Each global motion planner was configured with a maximum planning time of $T_{global}=0.2s$ . To account for the stochastic nature of the sampling-based planners, each planner performed 20 runs in each scenario, resulting in a total of 4,000 simulations. We employed several performance metrics, including the average, minimum, and maximum path length of the EE and the path duration for successful runs. A run was deemed successful if the robot reached the goal within 60s without colliding with any obstacles.

The results are presented in Table 1, with the best results highlighted in bold. Success rates highlighted in red indicate at least one run resulting in a collision. To facilitate cross-scenario performance comparison, we utilized a relative metric. This metric compares the results of each pre-planner against the shortest path length and shortest path duration for each scenario, with a minimum of 0% representing the best value across all scenarios. For instance, a value of 100% in the metric of maximum path length indicates that, on average across all environments, the longest paths generated by the pre-planner were twice as long as the respective shortest paths.

**TABLE 1:** Pre-planner comparison.

Pre-planner	Path length [%]			Success [%]	Path duration [%]		
	Mean	Max	Min		Mean	Max	Min
RRT LaValle (1998)	6.13	17.84	3.67	96.5	5.51	47.70	4.86
RRTConnect Kuffner and LaValle (2000)	4.42	19.52	1.93	98.0	4.33	23.09	5.06
RRT* Karaman and Frazzoli (2011)	4.28	**8.54**	3.66	96.5	**3.37**	**9.47**	6.67
TRRT Jaillet et al. (2010)	3.20	14.20	2.23	98.5	6.53	52.99	6.57
BiTRRT Devaurs et al. (2013)	4.93	22.44	3.44	96.5	7.90	54.90	8.44
LBT-RRT Salzman and Halperin (2016)	6.79	23.00	3.34	97.5	13.05	73.88	4.86
SBL Sánchez and Latombe (2003)	4.15	11.11	1.97	96.0	4.42	30.65	5.84
EST Hsu et al. (1997)	**2.20**	10.80	2.82	98.0	3.87	25.61	6.66
BiEST Hsu et al. (1997)	4.13	13.99	2.28	96.5	3.39	27.78	5.53
ProjEST Hsu et al. (1997)	4.14	11.81	1.55	98.0	4.70	32.28	5.60
KPIECE Şucan and Kavraki (2009)	3.18	10.81	2.23	97.5	5.40	44.85	7.13
BKPIECE Şucan and Kavraki (2009)	5.50	27.99	3.73	96.0	4.88	26.26	5.95
LBKPIECE Şucan and Kavraki (2009); Bohlin and Kavraki (2000)	3.76	10.72	3.22	96.5	6.05	53.49	7.37
PDST Ladd and Kavraki (2005)	4.61	18.92	2.50	97.5	5.82	46.03	6.77
STRIDE Gipson et al. (2013)	5.77	18.23	**0.91**	97.0	3.79	14.01	5.88
SPARS Dobson et al. (2013)	5.37	15.64	3.39	98.5	4.37	21.62	6.84
SPARS2 Dobson and Bekris (2013)	5.71	21.42	3.82	98.5	7.17	55.44	6.16
PRM Kavraki et al. (1996)	3.90	13.78	1.90	**100.0**	3.86	37.36	**3.94**
PRM* Karaman and Frazzoli (2011)	4.60	20.82	2.82	98.0	9.96	93.10	8.08
LazyPRM* Bohlin and Kavraki (2000); Karaman and Frazzoli (2011)	4.63	16.80	2.90	98.0	4.83	35.62	6.33

1. Bold values highlight the best results across all methods. 2. Red values indicate at least one occurrence of a collision during the respective run.

The absolute values of the performance metrics for each individual scenario and planner and illustrations of all scenarios are presented in the accompanying repository Becker et al. (2024). As observed in Table 1, the performance variation among different pre-planners is not substantial. This can be attributed to the predictive agent simulation of the ICF planner, which filters out suboptimal choices for the magnetic field vector. For further evaluations and comparisons with other motion planners, we selected two well-performing planners: PRM, owing to its flawless success rate, and RRT*, which exhibited the most best values.

Note that the pre-planner comparisons presented here were performed using a slightly different calculation method described in detail in Becker et al. (2023a). However, we only conduct a relative comparison to identify suitable pre-planners. Therefore, the modifications introduced in this chapter do not affect the relative performances, which we also verified in empirical tests.

### 3.2 Comparisons to other motion planners

**FIGURE 8:**
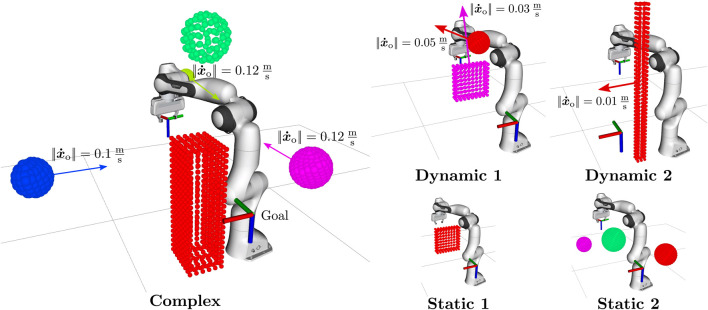
Simulation environments used for comparisons. Figure adapted from Becker et al. (2023a) (CC-BY-NC-ND).

Next, we compare our ICF planner in combination with the chosen pre-planners against other reactive and global motion planning approaches in five additional challenging environments, which are illustrated in Figure 8.

The reactive approaches considered in the following comparative analysis include the APF approach from Khatib (1986), the CF approach from Ataka et al. (2018b) and our extension of the CFP framework for robotic manipulators introduced in Becker et al. (2021). In order to enable a fair comparison of the force command generation, our implementations of the locally reactive controllers integrate our safety-improving fallback method from Section 2.2.4, and our definition of the attractive force from Section 2.1.1. We also compare our planning approach against the global sampling-based planners PRM (Kavraki et al., 1996), RRT* (Karaman and Frazzoli, 2011) and RRTConnect (Kuffner and LaValle, 2000). For a comparison with optimization-based motion planers, we included the MoveIt! implementation of the stochastic trajectory optimization for motion planning (STOMP) planner from Kalakrishnan et al. (2011). The selection of these planners is based on their widespread use, good results in the previous pre-planner analysis, and availability in the MoveIt! framework.

The sampling-based planners and the STOMP planner exclusively consider static obstacles and are consequently tested only in the two static environments, in which a maximum planning time of 0.5s was allowed. If no solution was found in this time, the attempt was considered a failure. All non-deterministic planners were executed until ten successful runs were recorded for each planner in each environment. A path duration exceeding 60s is also considered a failure. Note that *iteration time* refers to the planning time for global planners (depicted with a * in Table 2) while it is used to specify the calculation time for a control reference for the reactive planners. Also note that the path duration depends highly on the allowed maximum velocities. While the EE velocity of the force-based methods can be specified via a Cartesian maximum velocity, the random-based planners used here operate exclusively in joint space. Specifying an EE velocity by means of constraints in the joint space is not straightforward, therefore, a measurement of the duration for the affected planners was omitted.

**TABLE 2:** Motion planner comparison.

Env.	Method	Length [m]	Duration [s]	Success	Iter. Time [ms]
Static 1	PRM	1.81	-	100%	190.0*
	RRTConnect	1.51	-	100%	74.6*
	RRT*	1.71	-	100%	500.0*
	STOMP	**1.34**	-	100%	120.1*
	Khatib	1.65	17.81	True	0.020
	Ataka	1.86	19.56	True	**0.018**
	CFP	1.49	13.00	True	0.019
	ICF-PRM	1.41	11.88	100%	**0.018**
	ICF-RRT*	1.49	**11.78**	100%	0.019
Static 2	PRM	2.02	-	100%	237.3*
	RRTConnect	2.01	-	100%	257.8*
	RRT*	2.00	-	62%	500.0*
	STOMP	**1.72**	-	43%	163.3*
	Khatib	-	-	False	**0.039**
	Ataka	-	60.00	False	0.045
	CFP	-	-	False	0.209
	ICF-PRM	1.88	30.45	100%	0.057
	ICF-RRT*	2.15	**26.51**	100%	0.053
Dynamic 1	Khatib	1.50	11.46	True	**0.029**
	Ataka	1.53	10.87	True	0.030
	CFP	-	-	False	0.053
	ICF-PRM	**1.31**	**12.55**	100%	0.035
	ICF-RRT*	1.48	13.13	91%	0.033
Dynamic 2	Khatib	1.68	33.81	True	0.020
	Ataka	-	60.00	False	0.021
	CFP	**1.47**	12.75	True	0.029
	ICF-PRM	1.51	**11.96**	100%	0.016
	ICF-RRT*	1.54	12.55	100%	**0.014**
Complex	Khatib	-	-	False	0.082
	Ataka	-	-	False	0.080
	CFP	-	-	False	0.126
	ICF-PRM	**2.12**	**27.42**	71%	**0.048**
	ICF-RRT*	2.20	28.46	62%	0.058

1. Bold values highlight the best results across all methods. 2. Red values indicate at least one occurrence of a collision during the respective run. 3. Iteration times marked with an asterisk (*) correspond to the planning time of global planners. Unmarked iteration times denote the computation time for generating a control reference in reactive planners.

Overall, the results in Table 2 demonstrate that the ICF planner outperforms the other tested motion planners. Despite its path length in static environments being slightly larger than that of the optimizing STOMP planner, it remains within a comparable range while additionally being capable of reactively avoiding dynamic obstacles. The advantage of the ICF planner becomes particularly evident in the second static environment. Although the environment may not appear complicated at first, a relatively complex joint movement of the robot is required to avoid collisions with the obstacles. This complexity leads to challenges for the optimizing STOMP and RRT*, reflected in significantly lower success rates. The CFP planner also faced challenges in this environment due to the extensive number of predictive agents created, resulting in increased iteration times. Consequently, no suitable combination of avoidance directions could be found, and opposing forces on the control points led to a collision.

The *complex* environment (cf. Figure 8) requires simultaneous avoidance motions of the EE and the body, aiming to test the limits of the motion planners; our ICF planner was the only planner able to reach the goal successfully without a collision. However, as can be seen in Table 2, it did not succeed in all runs. The ICF planner failed when the pre-planner repeatedly could not find a path to the goal within the given planning time of 0.2s. In such cases, the ICF planner utilized the safety-improving fallback method, and the obstacles pushed the robot into configurations close to the joint limits, where no avoidance was possible, leading to an inevitable collision. Similarly, in one execution in the environment *Dynamic 1*, the ICF-RRT* planner ended up in a configuration close to the joint limits and was unable to reach the goal afterwards.

### 3.3 Human-robot interaction experiments

In this section, we demonstrate the capabilities of the planning framework in a real-world experimental setup with a Franka Emika Research 3 robot. The robot is programmed to move continuously between two predefined goal poses with a maximum velocity of 0.65 ms^-1^. The goal poses are updated when the robot is within a distance of 0.05 m of the current goal. During the execution of this task a human enters the workspace of the robot several times.

We use the body tracking feature of the Sterolabs ZED SDK^4^, which enables the identification and tracking of 18 keypoints on the human body. For the purposes of this experiment, we use a subset of these keypoints, specifically those corresponding to the upper body. These keypoints are used to create cylindrical approximations of the human torso, head, and arms, providing a simplified yet accurate representation of the dynamic obstacle. These approximations are then published in point cloud format, which is directly processed by our motion planning framework to ensure real-time adaptability. The interaction between the tracked human body and the robot’s movement can be observed in the accompanying video Becker et al. (2024), which shows how the cylindrical representations move in sync with the human collaborator.

**FIGURE 9:**
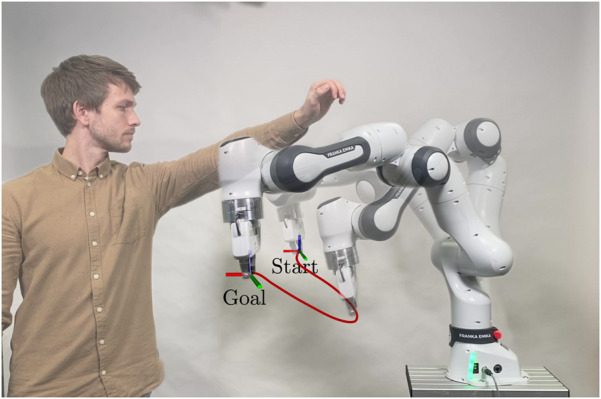
Excerpt from the experiment demonstrating the robot’s ability to avoid humans, as shown by the red path of its avoidance movement.

**FIGURE 10:**
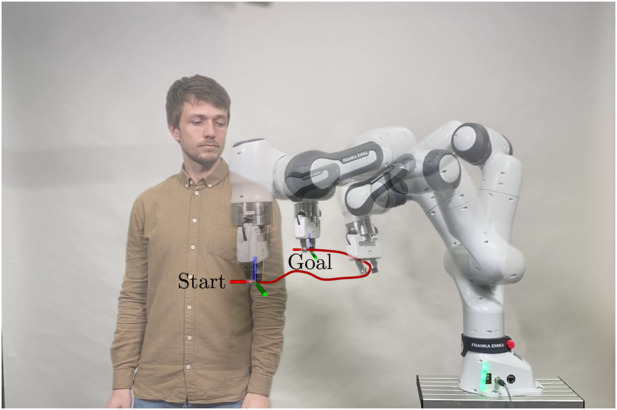
Excerpt from the experiment demonstrating the robot’s ability to avoid humans, as shown by the red path of its avoidance movement.

**FIGURE 11:**
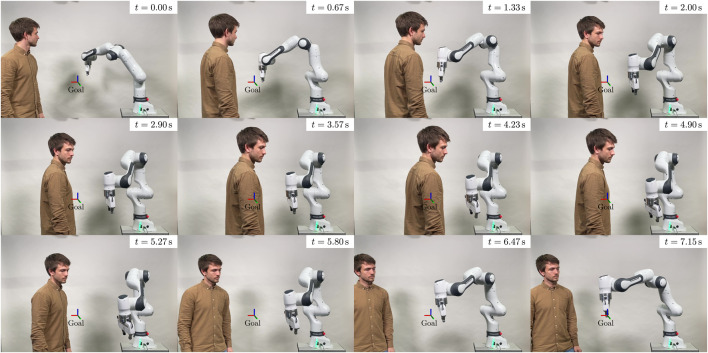
Excerpt from the experiment demonstrating the robot’s ability to avoid humans. The figure displays a sequence of timeframes where the human obstructs all potential paths to the goal pose, causing the robot to deviate from its original path.

The planner consistently demonstrates its ability to reactively avoid various dynamic motions of the human (cf. Figures 9, 10) even when the human obstructs all possible paths to the goal pose (cf. Figure 11).

## 4 Conclusion

In this paper, we introduced the ICF planning algorithm, which integrates reactive collision avoidance with global exploration by leveraging information about feasible avoidance directions from a global pre-planner. The ICF planner has been rigorously tested in a variety of simulations, demonstrating robust performance even in complex environments with multiple dynamic obstacles. Comparisons with other widely-used global and locally reactive motion planners demonstrate its superiority. Additionally, we validated the planning framework in a challenging real-world experiment, demonstrating its capability to react promptly and safely to fast movements of humans within its workspace. A promising extension could be to further exploit the high parallelizability of the planner by using multiple pre-planners simultaneously. In particular, a combination of fast global planners and optimizing planners with higher planning times is a promising extension. Future research should focus on improving the global information extraction module. Currently, it is unable to extract sufficient information when more complex obstacle avoidance motions are required.

## Data Availability

The datasets presented in this study can be found in online repositories. The names of the repository/repositories and accession number(s) can be found in the article/supplementary material.

## Footnotes

1
https://gitlab.com/roboterfabrik1/icf_planner

2 Note that this simplification is only possible when the planner is used to calculate a reference trajectory for a tracking controller that considers the robot dynamics. The actual inertia matrix M(q) should be used when the resulting torque command $\tau_{\text{ee}}$ is directly applied to the robot or when used in combination with any torque-based control interface.

3
https://github.com/stereolabs/zed-ros-wrapper

4
https://www.stereolabs.com/docs/body-tracking
